# The involvement of specific t cells in the pathogenesis of metamizole-induced agranulocytosis

**DOI:** 10.1186/2045-7022-4-S3-P107

**Published:** 2014-07-18

**Authors:** Jacqueline Adam, Antonia Bünter, Werner J Pichler, Thomas Wendland

**Affiliations:** 1 Clinic for Rheumatology and clin. Immunology/Allergology, University Hospital of Bern, Switzerland; 2 Adverse Drug Reactions – Analysis and Consulting GmbH, ADR-AC GmbH, Switzerland; 3 Clinic for internal medicine, University Hospital of Bern, Switzerland

## Background

Non-chemotherapy related, drug-induced agranulocytosis is a rare idiosyncratic reaction, which may be fatal. Along with betalactames, the analgesic metamizole is reported to occasionally cause agranulocytosis. The disease results in a severe reduction of granulocytes rendering affected patients susceptible to bacterial and fungal infections. The pathogenesis of drug-induced agranulocytosis is complex as non-immune (mainly toxic) and immune mechanisms might be involved.

## Aim

The identification and characterization of metamizole-specific T cells in patients with drug-induced agranulocytosis by generating metamizole-specific T cell lines.

## Methods

PBMCs from metamizole-allergic and metamizole-tolerant subjects were induced with 100ug/ml metamizole. Cultures were supplemented with IL-2 and were restimulated every 14 days. Drug-specific cell activation was determined by flow cytometry after a 6h restimulation phase.

## Results

After two restimulation rounds, metamizole-specific T cells were identified in a metamizole-allergic and in a metamizole-tolerant individual. In both cases, CD8+ T cells but no CD4+ cells reacted to the drug. Reactive cells secreted IFN and upregulated CD107a upon drug exposure. Interestingly, T cells were activated exclusively by metamizole in solution. Autologous antigen presenting cells incubated overnight in 100ug/ml metamizole prior to restimulation failed to activate CD8+ T cells. Furthermore, metamizole-specific T cells from both donors, allergic and tolerant, were self-reactive, i.e. reacted to the drug even in the absence of antigen presenting cells.

## Conclusion

The observed CD8+ T cell reactivity which was exclusively restricted to metamizole in solution supports a T cell activation according to the p-i concept (pharmacological interactions with immune receptors). Other hypersensitivity reactions underlying this concept revealed the crucial role of particular HLA class I molecules (e.g. HLA-B*57:01 in abacavir hypersensitivity). We therefore plan to type the study participants for their HLA class I expression in order to identify HLA molecules potentially involved in metamizole-induced agranulocytosis. This screening process will be supported by in silico modeling of metamizole binding to a panel of common HLA class I molecules. Furthermore, we plan to characterize metamizole-specific T cells in regards to their cytotoxic potential, i.e their expression of Granzyme B, Granulysin, perforin and FasL upon drug exposure.

